# MicroID2: A Novel Biotin Ligase Enables Rapid Proximity-Dependent Proteomics

**DOI:** 10.1016/j.mcpro.2022.100256

**Published:** 2022-06-08

**Authors:** Benjamin S. Johnson, Lexie Chafin, Daniela Farkas, Jessica Adair, Ajit Elhance, Laszlo Farkas, Joseph S. Bednash, James D. Londino

**Affiliations:** Department of Internal Medicine, Division of Pulmonary, Critical Care, and Sleep Medicine, Davis Heart and Lung Research Institute, The Ohio State University Wexner Medical Center, Columbus, OH, USA

**Keywords:** proximity labeling, BioID, TurboID, organelle, mass spectrometry, AA, amino acid, DMEM, Dulbecco's modified Eagle's medium, FDR, false discovery rate, HA, hemagglutinin, HRP, horseradish peroxidase, IFNGR1, interferon-gamma receptor 1, IFNLR1, interferon-lambda receptor 1, lbMicroID2, low background MicroID2, MS, mass spectrometry, MS/MS, tandem mass spectrometry, MTS, mitochondrial targeting sequence, NES, nuclear export sequence, NLS, nuclear localization sequence, PPI, protein–protein interaction, RRID, Research Resource Identifier, RT, room temperature

## Abstract

Identifying protein–protein and other proximal interactions is central to dissecting signaling and regulatory processes in cells. BioID is a proximity-dependent biotinylation method that uses an “abortive” biotin ligase to detect proximal interactions in cells in a highly reproducible manner. Recent advancements in proximity-dependent biotinylation tools have improved efficiency and timing of labeling, allowing for measurement of interactions on a cellular timescale. However, issues of size, stability, and background labeling of these constructs persist. Here we modified the structure of BioID2, derived from *Aquifex aeolicus* BirA, to create a smaller, highly active, biotin ligase that we named MicroID2. Truncation of the C terrminus of BioID2 and addition of mutations to alleviate blockage of biotin/ATP binding at the active site of BioID2 resulted in a smaller and highly active construct with lower background labeling. Several additional point mutations improved the function of our modified MicroID2 construct compared with BioID2 and other biotin ligases, including TurboID and miniTurbo. MicroID2 is the smallest biotin ligase reported so far (180 amino acids [AAs] for MicroID2 *versus* 257 AAs for miniTurbo and 338 AAs for TurboID), yet it demonstrates only slightly less labeling activity than TurboID and outperforms miniTurbo. MicroID2 also had lower background labeling than TurboID. For experiments where precise temporal control of labeling is essential, we in addition developed a MicroID2 mutant, termed lbMicroID2 (low background MicroID2), that has lower labeling efficiency but significantly reduced biotin scavenging compared with BioID2. Finally, we demonstrate utility of MicroID2 in mass spectrometry experiments by localizing MicroID2 constructs to subcellular organelles and measuring proximal interactions.

## Highlights

• MicroID2 is smallest biotin ligase yet described that facilitates robust labeling of proximal proteins.
• Low background MicroID2 allows slower labeling with less background biotinylation.
• MicroID2 can be utilized as a tool for proximity proteomics in subcellular organelles.

## Graphical Abstract

**Figure:**
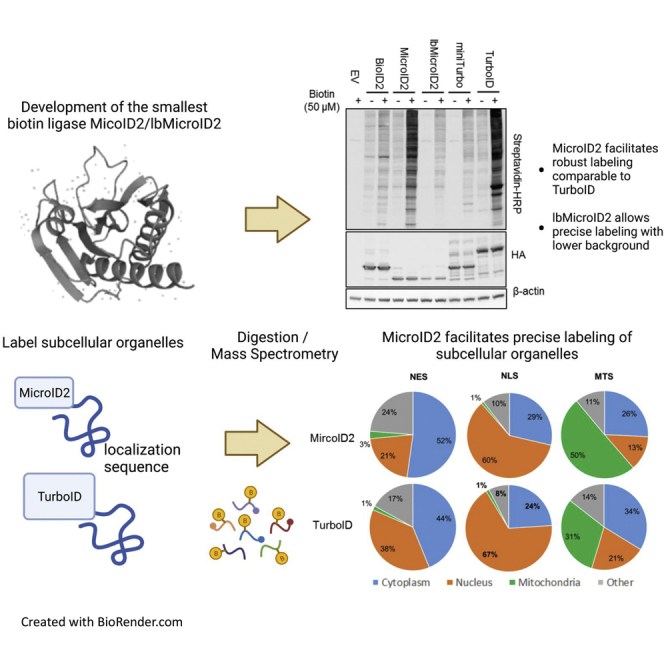

## In Brief

BioID uses a promiscuous biotin ligase to detect protein–protein interactions in a highly reproducible manner. Issues of size, stability, and background labeling of these constructs persist. Here, we modified the structure of BioID2 to create MicroID2, the smallest biotin ligase described. MicroID2 has comparable labeling to TurboID and outperforms miniTurbo. We also developed lbMicroID2, a mutant with lower labeling and less background. Finally, we demonstrated the utility of MicroID2 in mass spectrometry by measuring examining proteins in subcellular organelles.

One major objective of modern molecular biology is understanding protein interactomes. Identifying protein proximal interactions is central to investigating such protein networks to better understand cellular and molecular signaling pathways. Traditionally, affinity purification mass spectrometry (MS) has been utilized to measure protein–protein interactions (PPIs). However, this technique requires precise lysis conditions to maintain and detect PPIs, may miss weaker interactions, and can be technically challenging (1, 2). The proximity-dependent biotin identification method utilizes an “abortive” biotin ligase to covalently label proteins proximal to the protein of interest and allows the detection of protein interactions in a highly reproducible manner. The two most popular proximity-dependent biotinylation techniques are BioID and APEX (2, 3). BioID and APEX offer distinct advantages: APEX2 labels proteins quite rapidly (∼30 s) but requires the addition of high concentrations of hydrogen peroxide that may alter the intracellular environment (4, 5). BioID labels protein interactions with slower kinetics (typically 16–24 h for the first generation, minutes to hours for more recently developed constructs) and labels interactors after the addition of biotin (2, 5, 6). In addition, BioID has been demonstrated both *in vitro* and *in vivo* (7, 8).

**Fig. 1:**
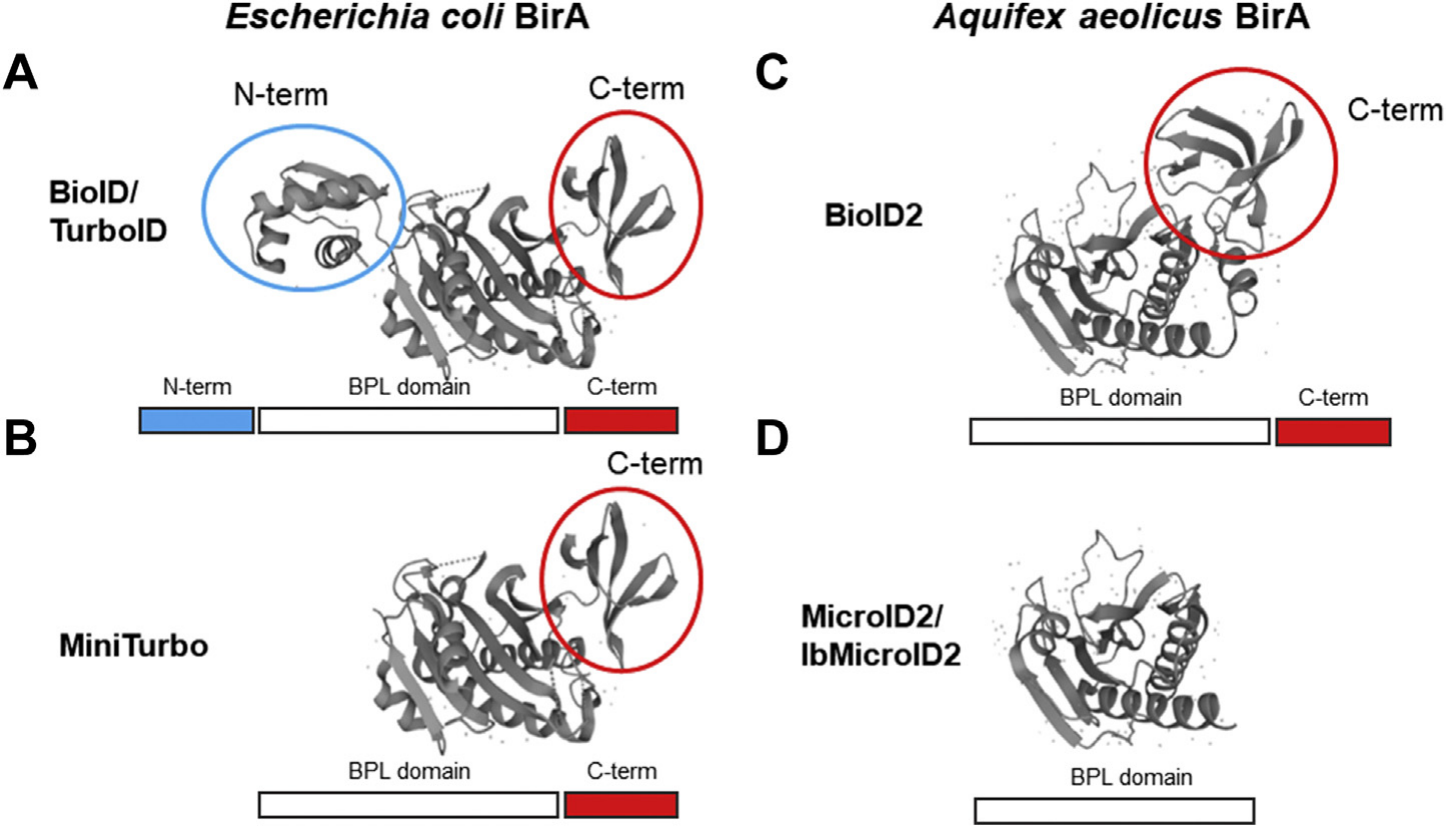
**Structure of biotin ligases used for BioD proximity-dependent biotinylation mass spectrometry.***A*, crystal structure of *Escherichia coli* BirA (Protein Data Bank [PDB] ID: 1BIA). *B*, crystal structure of *E. coli* BirA (PDB ID: 1BIA) with the N-terminal domain removed to resemble the miniTurbo structure. https://www.uniprot.org/uniprot/P06709. *C*, crystal structure of *Aquifex aeolicus* BirA (PDB ID: 2EAY) https://www.uniprot.org/uniprot/O66837. *D*, crystal structure of *A. aeolicus* BirA (PDB ID: 2EAY) with the truncated C-terminal domain to resemble MicroID2. BPL, biotin protein ligase domain.

All biotin protein ligases have a conserved central catalytic domain responsible for protein biotinylation. This catalytic site binds biotin and ATP facilitating the production of biotinyl-5′-AMP. Mutating the arginine 118 of *Escherichia coli* BirA to glycine led to a 100-fold greater *K*_*d*_ for biotin and a 400-fold higher dissociation rate for biotinyl-5′-AMP, allowing proximity-dependent biotinylation of neighboring proteins. More recently, the *Aquifex aeolicus* BirA was mutated in a similar fashion to produce a smaller biotin ligase named BioID2 (9). The smaller size and similar labeling efficiency of BioID2 is advantageous in situations where larger adducts would interfere with protein function. Next, the *Bacillus subtilis* BirA (BASU) was developed and demonstrated increased labeling efficiency over the original BioID (10). Most recently, the *E. coli* BirA BioID enzyme was optimized by directed evolution for dramatically faster labeling, yielding TurboID and miniTurbo (Fig. 1) (11). While TurboID is highly active, its propensity to initiate labeling from low levels of biotin already in media can result in high signal-to-noise ratio and even toxicity in some *in vitro* and *in vivo* models (11, 12). While many cell culture media formulations contain biotin, even using biotin-free formulations (Dulbecco's modified Eagle's medium [DMEM] and Eagle's minimal essential medium) and dialyzed serum does not completely eliminate background labeling (9). MiniTurbo has reduced background labeling but is less active overall and demonstrates instability under some circumstances such as when stably integrated into a human cell line or when expressed in the adult worm or adult fly (11, 12).

Despite the advantages of proximity-dependent biotinylation compared with alternative identification techniques, currently available biotin ligases suffer from large size, instability under certain conditions, and high background. Here, we report on a new construct called “MicroID2,” developed through rational modification of BioID2 to create a smaller and faster biotin-labeling ligase. We demonstrate that truncation of the C terminus of BioID2 increased labeling efficiency, whereas simultaneously decreasing background biotinylation. We optimized the construct for maximal activity and low-background biotinylation prior to exogenous biotin. The new construct, MicroID2, has lower background labeling than the original BioID2 with a robust increase in biotin-induced labeling. MicroID2 is the smallest proximity ligase described at ∼19 kD with labeling activity slightly lower than TurboID (∼37 kD) but higher than miniTurbo (∼27 kD) (Fig. 1, *A*–*D*). In addition, we developed a MicroID2 mutant with lower background labeling that can be utilized in situations where discriminating between background and true interactors is challenging (*i.e.*, in cell culture systems that require low quantities of biotin and in *in vivo* systems). Both these ligases provide important advantages for the execution of proximity-dependent biotinylation experiments.

## Experimental Procedures

### Cell Culture

Human embryonic kidney 293FT cells (American Type Culture Collection) were cultured with DMEM containing 10% fetal bovine serum and antibiotics. BEAS-2B cells were maintained with hydrocortisone, insulin, transferrin, estradiol, and selenium added to DMEM/F12 medium) supplemented with 10% fetal bovine serum. Stable BEAS-2B cells expressing MicroID2 and TurboID constructs were maintained in hydrocortisone, insulin, transferrin, estradiol, and selenium supplemented with puromycin (2 μg/ml)

### Plasmids, Cloning, and Transfection

Several biotin ligase constructs utilized for this study were obtained from Addgene, including MCS-BioID2-HA (plasmid no.: 74224), FLAG-TurboID (plasmid no.: 124646), and V5-miniTurbo-NES_pCDNA3 (plasmid no.: 107170). Plasmids were transfected into cells using XtremeGene HP DNA transfection reagent (Roche, Applied Science) according to the manufacturers' protocols.

### Stable Integration With Sleeping Beauty Transposase System

MicroID2 and TurboID constructs were cloned into the sleeping beauty backbone pSBbi-RB (Addgene; plasmid no.: 60522). Site-directed mutagenesis or restriction enzyme cloning was utilized to introduce localization sites: 3× nuclear localization sequence (NLS) (Addgene; plasmid no.: 98875), mitochondrial targeting sequence (MTS) (Addgene; plasmid no.: 98876), and the nuclear export sequence (NES) (Addgene; plasmid no.: 49386). The final constructs were transfected into BEAS-2B and cotransfected with the sleeping beauty transposase (pCMV(CAT)T7-SB100; Addgene; plasmid no.: 34879) at a 1:5 ratio. At 4 to 5 days post-transfection, cells were selected by puromycin A1 treatment (2 μg/ml). After selection, integration was confirmed by measuring FLAG expression and biotinylation *via* immunofluorescence and Western blotting.

### Immunoblotting

Immunoblotting was performed as previously described (13). Cells were lysed in radioimmunoprecipitation assay buffer, sonicated, and clarified by centrifugation. Lysates were diluted in SDS protein sample buffer. Proteins were separated by electrophoresis and transferred to a nitrocellulose membrane. Blots were blocked in 5% milk, followed by probing overnight with antibodies followed by washes with Tris-buffered saline with 0.1% Tween. For streptavidin–horseradish peroxidase (HRP) washes, Tris-buffered saline containing 0.1% Tween, 0.25 M NaCl, and 0.2% SDS was utilized to decrease background. Following addition of secondary antibodies (goat antimouse; Bio-Rad, catalog no.: 170-6516; goat anti-rabbit; Bio-Rad, catalog no.: 170-6515; 1:2000 dilution), membranes were developed using a Western Bright Sirius immunoblotting detection kit (Advansta) and imaged using Bio-Rad Image Lab. Single band intensity was quantified using ImageJ (NIH; https://imagej.nih.gov/ij/) software. Antibodies: β-actin (catalog no.: A5441; Sigma), V5 (catalog no.: R960–25; Fisher Scientific), hemagglutinin (HA; ANTI-HA DW2; Sigma), FKBP (catalog no.: 55104; Cell Signaling), FRB (mammalian target of rapamycin human FRB domain; ENZO), FLAG (ANTI FLAG M2; Fisher), and streptavidin–HRP (catalog no.: 405210; BioLegend).

### Immunofluorescence

Immunofluorescence was performed as described previously (14). Briefly, cells were plated and grown to confluence on glass bottom culture slides. The cells were washed with PBS, fixed with 2% paraformaldehyde for 15 min, permeabilized with 0.1% Triton X-100, blocked with 2% bovine serum albumin for 1 h, and then exposed to 1:250 primary antibodies overnight at 4 °C, followed by incubation with Alexa Fluor–conjugated secondary antibodies (Thermo Fisher Scientific; 1:1000 dilution) and then briefly stained with 4′,6-diamidino-2-phenylindole at room temperature (RT). Cells were imaged using the Olympus FV 3000 Confocal System (Olympus Life Sciences).

### Biotinylation

The biotinylation procedure was performed as previously described with modifications (15). Transiently transfected cells were treated with biotin (D Biotin; Fisher) at the indicated times and concentrations. For the isolation of biotinylated proteins, the biotin-containing media was replaced with biotin-free media 10 min prior to harvest. Cells were then lysed in buffer A (0.5% Triton X-100 and 0.2% SDS), sonicated, and clarified by centrifugation. Biotinylated proteins were isolated *via* pulldown with Pierce Streptavidin Magnetic Beads (Thermo). Samples were washed twice with 1 ml wash buffer 1 (1% Triton and 0.2% SDS) at RT for 8 min on the rotator. Samples were then washed twice with wash buffer (8 M urea in 50 mM Tris, pH 7.4; prepared fresh for experiment) for 8 min on a rotator at RT, followed by a final wash with 1× wash buffer 1 at RT for 8 min on rotator. Beads containing proteins were either submitted for MS experiments or eluted with 2× SDS buffer + 1 mM biotin for Western blotting.

### MS

Streptavidin beads were washed with 50 mM ammonium bicarbonate three times and incubated with sequencing grade–modified trypsin (Promega) at 37 °C overnight, and beads were vortexed twice during the incubation for 30 s. The reaction was quenched the next morning by adding acetic acid for acidification. Supernatant was concentrated for LC/MS–MS analysis. Tandem mass spectrometry (MS/MS) was performed on a Thermo Fisher Scientific Orbitrap Fusion mass spectrometer equipped with a Nanospray FAIMS Pro Sources operated in positive ion mode. Tryptic peptides (4.0 μl) were separated on an Easy-Spray nano column (Pepmap RSLC, C18 3 μm, 100 Å, 75 μm × 150 mm; Thermo Fisher Scientific) with increasing organic over 120 min at a flow rate of 300 nl/min. Three FAIMS compensation voltages (−50, −65, and −80 V) were used for data acquisition. Data were searched using Mascot Daemon by Matrix Science, version 2.7.0 *via* ProteomeDiscoverer (version 2.4; Thermo Fisher Scientific). Mascot was set up to search the Common_Contamination_Proteins-cRAP_LZ.fasta and UniProt Human database (20210604; 20,513 entries) assuming the digestion enzyme “stricttrypsin” as a parameter during the database search. Mascot was searched with a fragment ion mass tolerance of 0.50 Da and a parent ion tolerance of 10.0 ppm. Carbamidomethylation of cysteine was specified in Mascot as a fixed modification. Deamidation of asparagine and glutamine and oxidation of methionine were specified in Mascot as variable modifications. Four missed cleavages for the enzyme were permitted. Only peptides with probability ≥95% will be considered as valid identification. Scaffold (version Scaffold_4.11.0; Proteome Software, Inc) was also used to validate MS/MS-based peptide and protein identifications. Peptide identifications were accepted if they could be established at greater than 96.0% probability to achieve a false discovery rate (FDR) less than 1.0% by the Scaffold Local FDR algorithm. Reliable protein identifications, containing at least two unique peptides, were accepted if they could be established at greater than 99.0% probability to achieve an FDR less than 1.0%; probabilities were assigned by the Protein Prophet algorithm (16). Proteins that contained similar peptides and could not be differentiated based on MS/MS analysis alone were grouped to satisfy the principles of parsimony. Label-free quantitation was performed using the spectral count approach, in which the relative protein quantitation is measured by comparing the number of MS/MS spectra identified from the same protein across samples.

### Experimental Design and Statistical Rationale

For the Western blotting experiments examining biotin ligase activity of truncated BioID2 and the G40S mutant, each experiment was performed in two independent experiments. Experiments examining substitution in the BioID2 active site were also performed in two independent experiments. Experiments examining substitution of lysine residues in the active site were performed in three independent experiments and quantified *via* densitometry. Experiments examining the substitution of residues in a highly conserved site were performed in two independent experiments. We then compared each of the biotin ligase constructs to BioID2, miniTurbo, and TurboID both alone and conjugated to a protein that was not previously tested during the derivation of MicroID2. Each experiment was performed in three independent experiments and quantified by densitometry. In all, our novel MicroID2 and lbMicroID2 (low background MicroID2) constructs were tested in multiple independent experiments and in three separate contexts (alone or conjugated to two distinct proteins). Quantification of multiple samples was performed using one-way ANOVA followed by Tukey's *t* test. Immunofluorescence was performed in two independent experiments, and representative images were selected. For proteomics analysis, we submitted 18 total samples (n = 3 samples [separate plates derived from the integrated cell lines, treated, and processed independently], n = 3 constructs; n = 2 biotin ligases).

## Results

### Deletion of the C Terminus of BioID2 Increases Activity and Inducibility

**Fig. 2:**
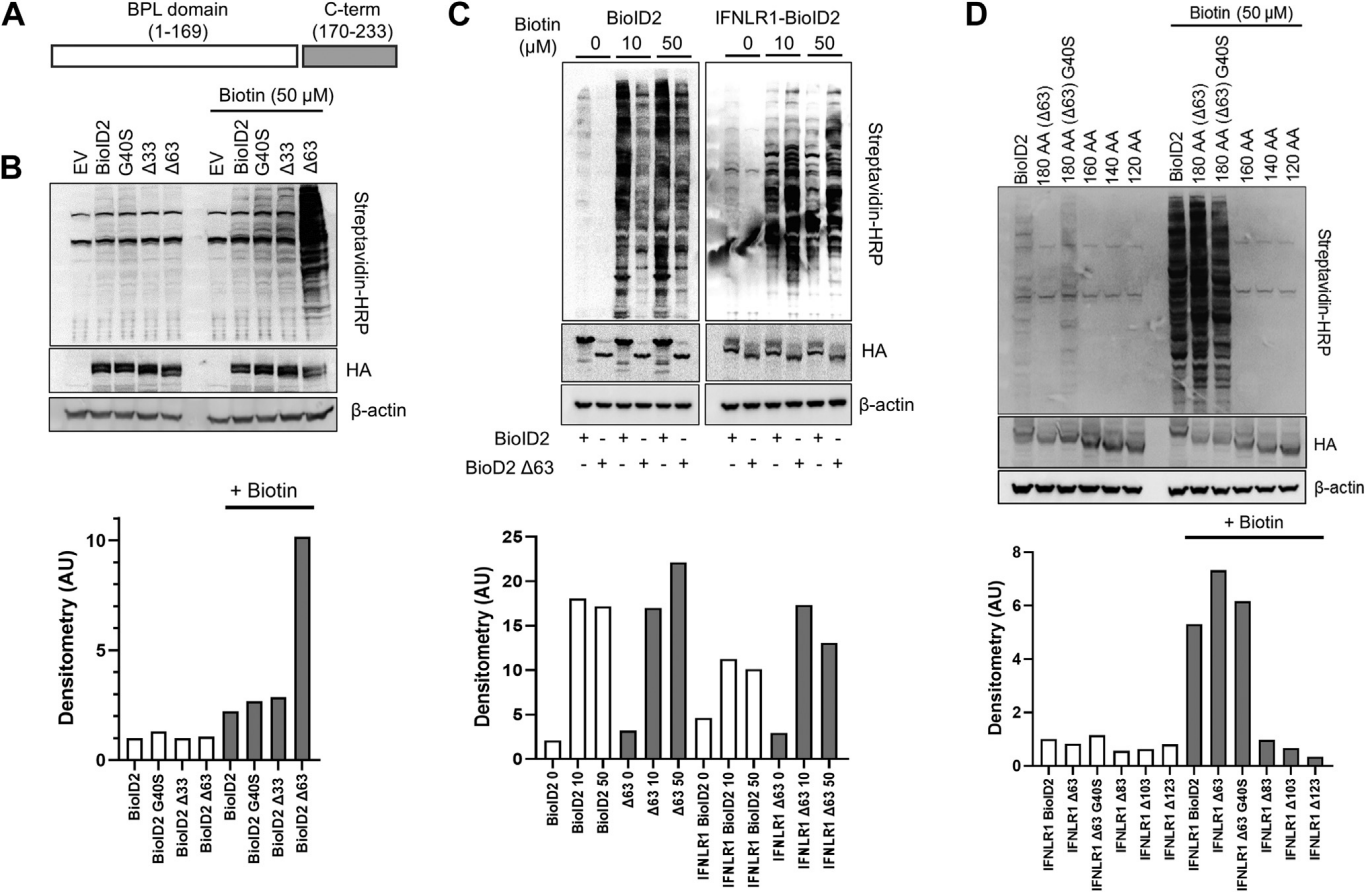
**Deletion of the C terminus of BioID2 increases biotin labeling.***A*, schematic depicting the C-terminal biotin protein ligase (BPL) domain and the C-terminal domain. *B*, human embryonic kidney (HEK) cells were transiently transfected with the following constructs: IFNGR1 conjugated to BioID2 (BioID2), G40S-BioID2 (G40S), Δ33-BioID2 (Δ33), and Δ63-BioID2 (Δ63). About 48 h post-transfection, cells were treated with biotin for 24 h and harvested at 72 h post-transfection. HA-tagged BioID2 expression was determined using anti-HA antibody. Biotinylated proteins were detected with streptavidin-HRP. *Below*, densitometry of streptavidin normalized to HA expression. *C*, comparison of BioID2 *versus* truncated BioID2: HEK cells were transfected with unconjugated BioID2 or unconjugated BioID2 Δ63. In addition, cells were transfected with IFNLR1-BioID2 and IFNLR1-BioID2 Δ63. At 48 h post-transfection, cells were labeled for 24 h with biotin at the indicated concentrations and probed as described previously. *Below*, densitometry of streptavidin normalized to HA expression. *D*, examination of additional truncations of BioID2: HEK cells were transfected with the full-length BioID2 (BioID2) or additional constructs represent the first 120 to 180 AA of BioID2. At 48 h post-transfection, cells were labeled for 24 h with biotin at the indicated concentrations and probed as described previously. *Below*, densitometry of streptavidin normalized to HA expression. *B*–*D*, representative of at least two independent experiments. AA, amino acid; HA, hemagglutinin; HRP, horseradish peroxidase; IFNGR1, interferon-gamma receptor 1.

Unlike the BirA from *E. coli* from which BioID was derived, which contains the biotin protein ligase domain plus the N-terminal and C-terminal domains, the BirA from *A. aeolicus* only contains the C-terminal domain. We tested whether this domain was either partially or wholly dispensable for the activity of BioID2 (Figs. 1*C* and 2*A*). Because our laboratory was interested in proximal interactions of membrane proteins, we hypothesized that a truncated biotin ligase conjugated to a membrane protein may retain stability comparable to that of the original BioID2. Interestingly, when we appended truncated BioID2 to interferon-gamma receptor 1 (IFNGR1), a heterodimeric transmembrane receptor that signals through the Janus kinase—signal transducer and activator of transcription pathway. We found that deletion of the C-terminal 33 amino acids (AAs) and to a greater extent deletion of 63 AA increased the labeling efficiency as determined by total streptavidin-labeled protein *via* Western blot (Fig. 2*B*). Background labeling with the Δ63 mutant was not increased compared with the original BioID2. In the development of proximity-dependent biotinylation assays, bacterial biotin ligases were mutated to facilitate function in cell-based systems. The *E. coli* BirA was adapted by R118G mutation to create the BioID “abortive” biotin ligase. As the arginine residue occupies a conserved region in BirA, *A. aeolicus* BirA was similarly mutated at R40G to create BioID2 (9). Another study demonstrated that a different substitution of this arginine, R118S, enhanced the biotinylation activity of BioID compared with R118G (11). Similarly, we mutated the comparable G40 site to serine and measured labeling (Fig. 2*B*). G40S led to a modest increase in biotinylation but much less pronounced than our observations with the truncation mutants. To confirm that truncation-mediated enhancement of activity was not specific to IFNGR1, we measured the activity of BioID2 both alone and conjugated to similar protein in the same family, interferon-lambda receptor 1 (IFNLR1). Consistent with the results of IFNGR1-BioID2-Δ63, IFNLR1-BioID2-Δ63 had increased activity *versus* IFNLR1-BioID2 and lower background labeling. To determine if the truncated construct can function independently of the conjugated protein, we compared BioID2 to the BioID2-Δ63. BioID2-Δ63 was slightly less active than the full-sized BioID2 construct, but both constructs labeled with similar efficiently when normalized to total expression. As with the membrane-conjugated BioID2-Δ63, background labeling appeared reduced compared with the original BioID2, but this effect was lost when normalizing for expression of the biotin ligase (Fig. 2*C*).

To determine the optimal site for C-terminal truncation, we made additional truncations beyond Δ63. Further 20 AA truncations, Δ83, Δ103, and Δ123 completely inactivated the biotinylation activity (Fig. 2*D*), as did an additional 10 AA deletion (Δ73, supplemental Fig. S1). Deletion of 10 AAs from the N-terminal domain also completely inactivated the enzyme (supplemental Fig. S1). We also examined whether the G40S mutant would have an additive effect with Δ63 truncation. However, the G40S mutant not only had modestly decreased total biotinylation but also had increased the background labeling (Fig. 2*D*). Therefore, we moved ahead with the R40G-Δ63 construct. These data demonstrate that truncation of the biotin ligase BioID2 results in a small, highly active, biotin ligase with decreased background labeling.

### Substitutions in the BioID2 Active Site Increased Labeling Efficiency

**Fig. 3:**
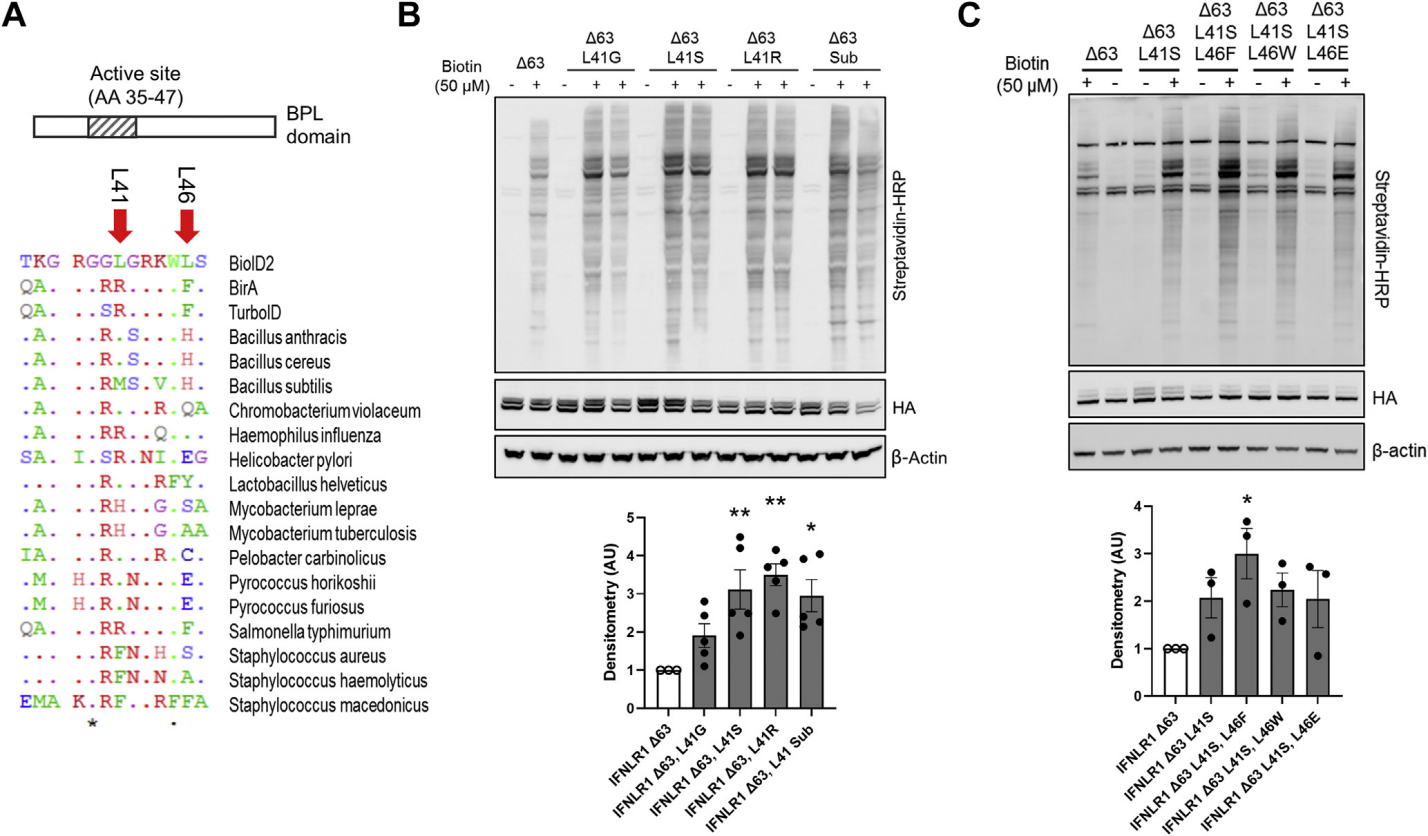
**Substitution in the BioID2 active site enhances biotin labeling.***A*, alignment of the active site of BioID2 (AA 35–47) using the GENtle software. *Arrows* indicate where substitutions examined occur in the active site. *B*, human embryonic kidney (HEK) cells were transfected with IFNLR1-BioID2 (Δ63) with mutations at the L41 site. Sub, AAs from the active site of *Bacillus subtilis* BirA. At 48 h post-transfection, cells were labeled for 2 h with biotin at the indicated concentrations and probed with streptavidin–HRP to measure biotinylation. *Below*, densitometry of streptavidin normalized to HA expression. *C*, HEK cells were transfected with IFNLR1-BioID2 mutants: Δ63, Δ63-L41S, Δ63-L41S-L46F, Δ63-L41S-L46E, and Δ63-L41S-L46W. At 48 h post-transfection, cells were labeled for 2 h with biotin at the indicated concentrations and probed with streptavidin–HRP to measure biotinylation. *Below*, densitometry of streptavidin normalized to HA expression. *B* and *C*, representative of three independent experiments. ∗*p* < 0.05, ∗∗*p* < 0.01 *versus* IFNLR1-Δ63 by ANOVA. AA, amino acid; HA, hemagglutinin; HRP, horseradish peroxidase; IFNLR1, interferon-lambda receptor 1.

A crystallographic study of *A. aeolicus* BirA found that the leucine 41 residue blocked biotin access to the biotin-binding site (17). This led us to speculate that mutation of this site (to alleviate the steric hindrance) would enhance labeling efficiency (Fig. 3*A*). We chose arginine as a replacement since it was highly conserved at the 41 site. We mutated to glycine to remove all side-chain interactions at the site. We also mutated to a serine residue to add a polar small AA substitution with a minimal steric hindrance that had previously been demonstrated to enhance activity in the active site (11). Finally, we examined how a more substantial change to the active site might alter activity. To do this, we made use of sequence alignments of the BirA protein of *B. subtilis* that has been demonstrated to be highly active when it was adapted as a biotin ligase called BASU (10). We examined whether substitution of the active site (AA 41–46; LGRKWLS-MSRVWH) would enhance IFNLR1-BioID2-Δ63 biotinylation activity (10). All mutants tested were considerably more active than the wildtype BioID2 active site. Based on these experiments and previous results, we chose to continue further testing with the L41S mutant (Fig. 3*B*). An additional observation of the crystal structure of *A. aeolicus* BirA found that the conserved L46 residue also changed conformation in the R40G mutant resulting in weak ATP binding in the active site. The consensus-conserved residue in this site primary consisted of bulky AA side chains. We replaced L46 with several substitutions. First, the mutation of glycine (L46G) completely inactivated biotinylation activity (supplemental Fig. S2). Replacement with the bulkiest side-chain tryptophan (L46W) did not change biotinylation appreciably. Mutation of L46 to the conserved glutamic acid (L46E) also did not change labeling. However, substitution to L46F did lead to a significant increase in labeling (Fig. 3*C*). We therefore continued our testing with the L41S, L46F mutant.

### Substitution of Lysine Residues in the Active Site Decreases Background Labeling

**Fig. 4:**
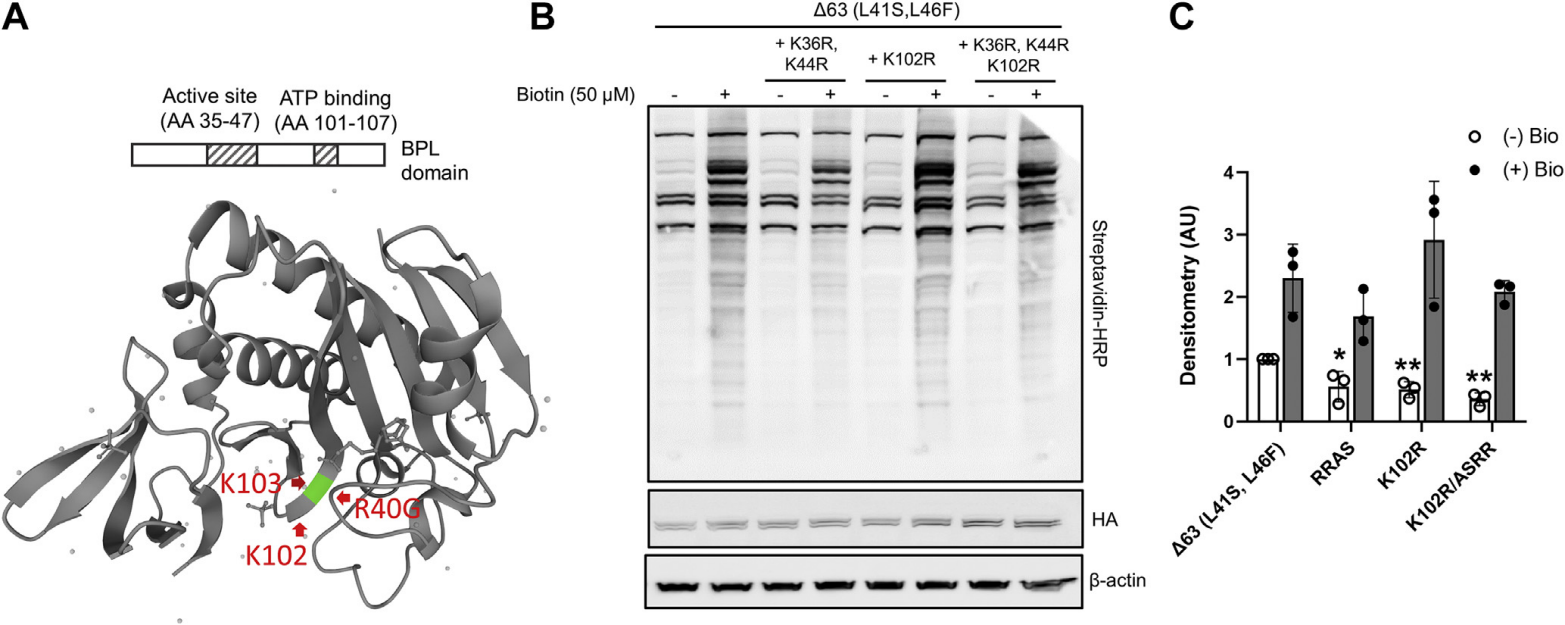
**Substitution of lysine residues in the active site moderately increases biotin labeling.***A*, crystal structure of *Aquifex aeolicus* BirA (PDB ID: 2EAY; https://www.uniprot.org/uniprot/O66837) with residues R40G, K102, and K103 depicted. *B*, human embryonic kidney (HEK) cells were transfected with IFNLR1-BioID2 (Δ63, L41S, and L46F) with additional mutations at K36, K44, and K102. At 48 h post-transfection, cells were labeled for 1 h with biotin at the indicated concentrations and probed as described previously. *C*, densitometry of biotin labeling *via* streptavidin–HRP normalized to HA expression. *B* and *C*, n = 3 experiments. ∗*p* < 0.05, ∗∗*p* < 0.01 *versus* IFNLR1-Δ63 (L41S, L46F) by ANOVA. HA, hemagglutinin; HRP, horseradish peroxidase; IFNLR1, interferon-lambda receptor 1; PDB, Protein Data Bank.

Because biotin is covalently attached to lysine residues, we speculated that lysine within the active site could be subject to self-biotinylation that might lead to altered biotin ligase activity (Figs. 4*A* and S3*A*). Mutation of lysines (K36, K44) within the active site showed a moderate reduction in both background labeling and biotin-induced labeling. Lysines 102 and 103 are also within the active site in the three-dimensional protein. While mutation of the highly conserved K103 to arginine severely inhibited biotinylation (supplemental Fig. S3*B*), mutation of K102 to arginine modestly, but not significantly, enhanced biotinylation without increasing background labeling (Fig. 4, *B* and *C*). The addition of the K36R, K44R, and K102R triple mutant resulted in approximately one-third of the background labeling of the Δ63 (L41S, L46F) construct with similar maximum labeling. Because total biotinylation was unchanged and background labeling was significantly decreased, we chose to include all three mutations.

### Mutation of a Highly Conserved Site Reduced Biotin Scavenging

**Fig. 5:**
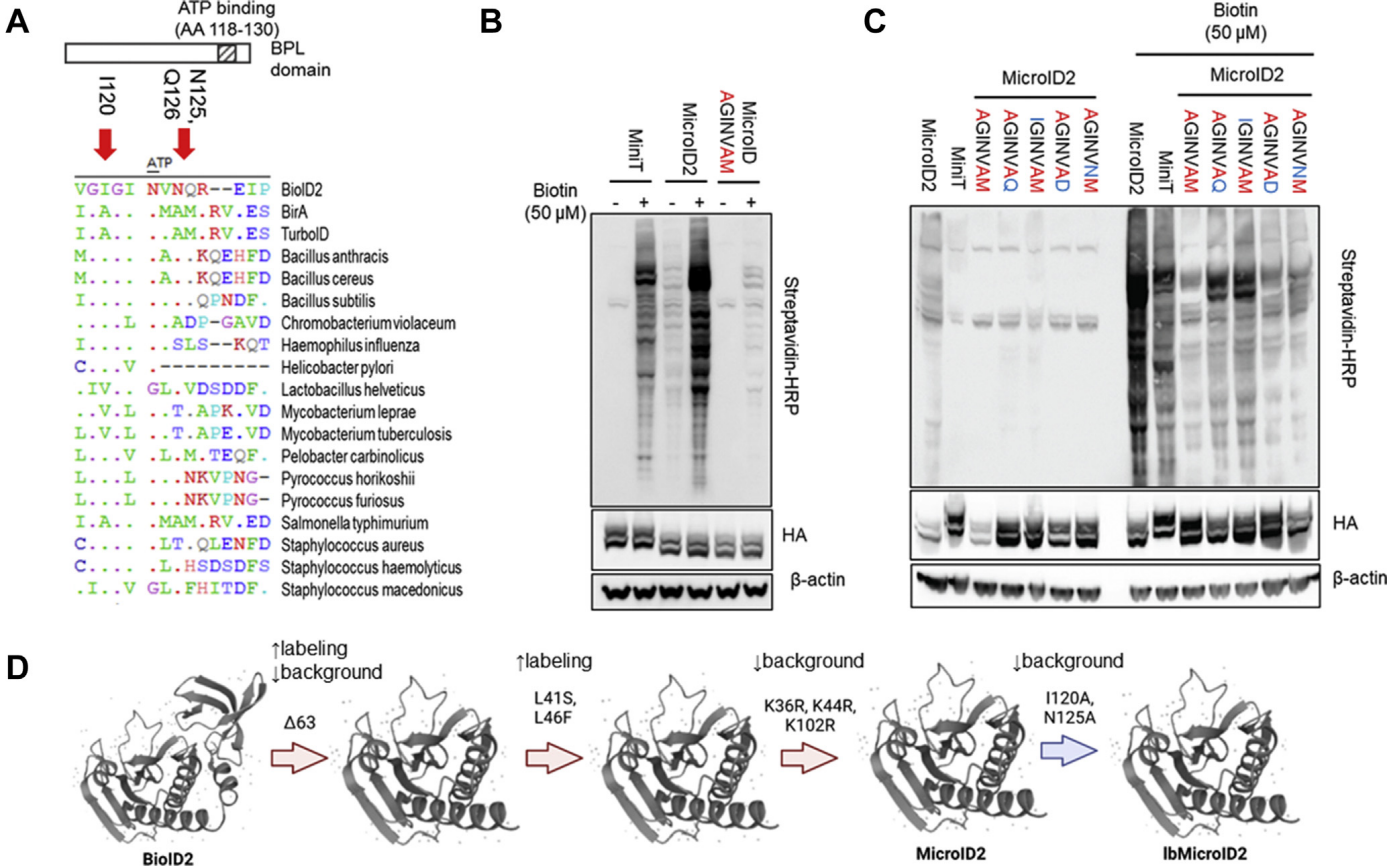
**Mutation of a highly conserved site reduced biotin scavenging.***A*, alignment of a conserved site of BioID2 (AA 118–130) using the GENtle software. *Arrows* indicate substitutions. N123 has previously been shown to bind ATP in the biotin–ATP. *B*, human embryonic kidney (HEK) cells were transfected with IFNLR1-miniTurbo, IFNLR1-MicroID2, and IFNLR1-MicroID2 mutated to resemble miniTurbo (AGINVAM) (altered residues in *red*). *C*, HEK cells were transfected with IFNLR1-MicroID2, IFNLR1-miniTurbo, IFNLR1-MicroID2 (AGINVAM), and IFNLR1-MicroID2 (AGINVAM) with restorative mutants (*blue*). At 48 h post-transfection, cells were labeled for 1 h with biotin at the indicated concentrations and probed as described previously. *D*, schematic depicting the deletions and substitutions introduced in the development of MicroID2 and lbMicroID2 and the consequences of each alteration. *B* and *C*, representative of at least two independent experiments. AA, amino acid; IFNLR1, interferon-lambda receptor 1.

MicroID2 is a highly active biotin ligase, which may be advantageous for measuring events at short time points in which high background labeling is detrimental. We also wanted to develop a mutant that had less biotin scavenging to limit background biotinylation. Because the miniTurbo construct had a reduced background labeling compared with BioID2, we speculated that differences in conserved sites may be responsible (11). Several residues in the conserved ATP-binding site at AA 120 to 126 of BioID2 were distinct from miniTurbo (identical to the TurboID sequence at this site) (Fig. 5*A*). Mutation of the MicroID2 IGINVNQ site to the miniTurbo AGINVAM not only significantly reduced biotinylation but also eliminated background labeling (Fig. 5*B*). To obtain a more favorable activity to background ratio, we reverted individual AAs to MicroID2. We found that mutating M125 back to Q (AGINVAQ) partially restored activity, while retaining low background labeling (Fig. 5*C*). This construct contains the substitutions I120A and, N124A in addition to the previously described MicroID2 substitutions, and was named lbMicroID2 to indicate the lower background labeling. This construct is beneficial in situations where a high signal-to-noise ratio impedes the ability to discriminate between background and induced labeling.

Having determined that the C-terminal domain was not essential for BioID2 activity, we speculated that the C-terminal domain of miniTurbo might also be dispensable. Based on the crystal structure of BirA, we deleted 43 AA of the C-terminal domain (supplemental Fig. S4*A*). Unlike MicroID2, deletion of the C-terminal domain completely inactivated miniTurbo (supplemental Fig. S4*B*). Because we found that AA 121 to 127 contributed to the high activity of MicroID2, we tested whether mutation of this region in the truncated miniTurbo to resemble MicroID2 (AGINVAM-AGINVNQ) could restore activity. However, this mutation was not sufficient to enhance the truncated miniTurbo activity to detectable levels (supplemental Fig. S4*B*). Therefore, dispensability of the C-terminal domain may be an inherent property of *A. aeolicus* BirA and perhaps other small biotin ligases from similar families.

The final constructs developed contain a 63 AA deletion at the C-terminal domain, mutation of the active-site residues L41S and L46F to increase biotinylation activity, and lysine to arginine mutations at K36, and K44, and K102 to prevent self-biotinylation. We named this new construct MicroID2 (Fig. 5*D*). Additional mutations I120A and N124A led to a ligase with lower background and less biotinylation activity. We named this construct lbMicroID2 (Fig. 5*D*).

### Comparison of Biotin Ligase Activity

**Fig. 6:**
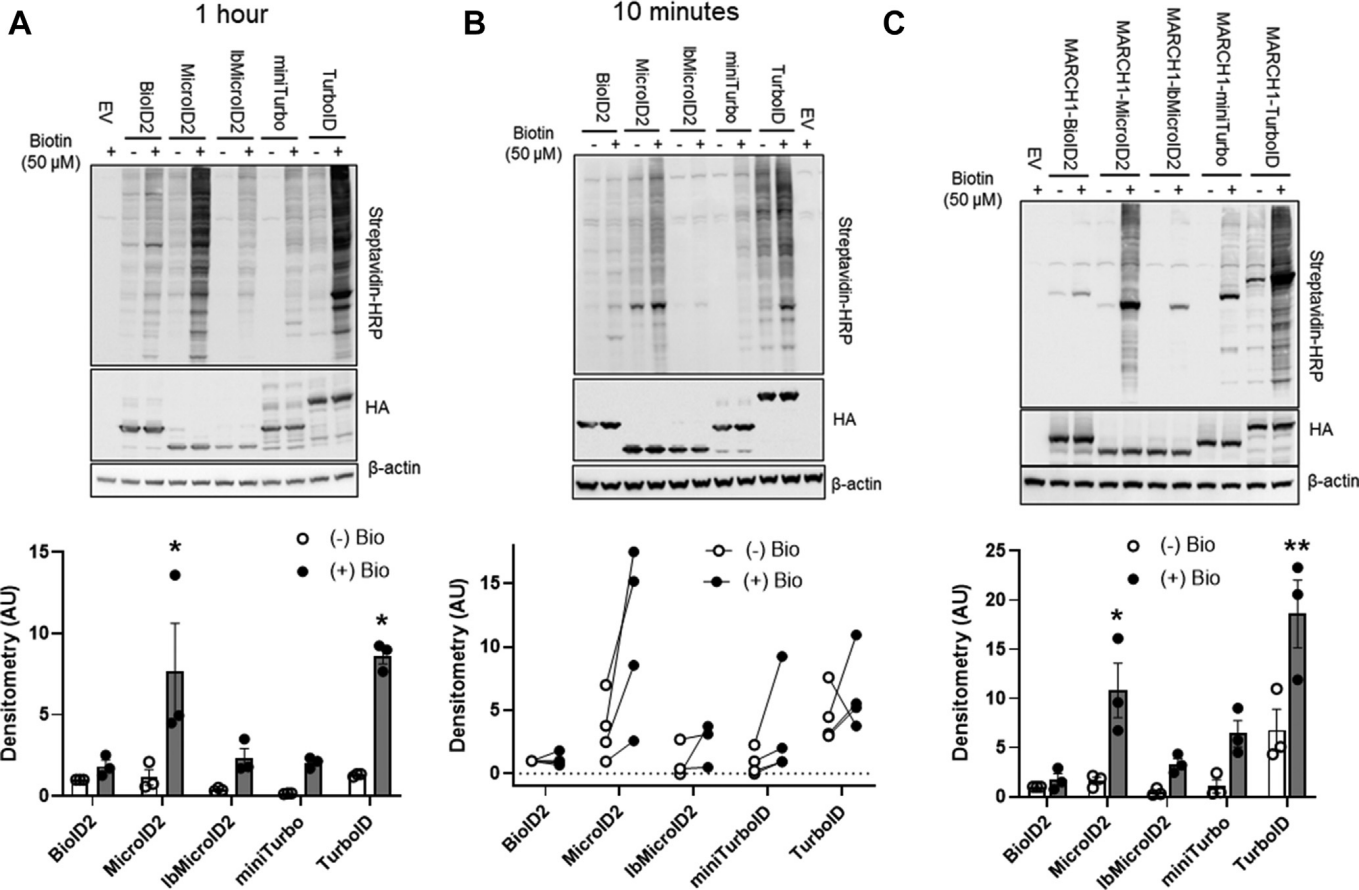
**Comparison of biotin ligase activity.** Human embryonic kidney (HEK) cells were transfected with several biotin ligases including BioID2, MicroID2, lbMicroID2 (containing additional I120A, N125A mutants), miniTurbo, and TurboID. Constructs were either expressed alone (*A* and *B*) or conjugated to the transmembrane protein MARCH1 (*C*). At 24 to 48 h post-transfection, cells were labeled with biotin for 1 h (*A* and *C*) or for 10 min (*B*), and cell lysates were harvested. HA-tagged biotin ligase expression was determined using anti-HA antibody. Streptavidin–HRP labels biotinylated proteins in the whole-cell supernatant. *Below*, densitometry of streptavidin normalized to HA expression with unconjugated (*A* and *B*) and the MARCH1-conjugated biotin ligases (*C*). *A*–*C*, n = 3 experiments, ∗*p* < 0.05, ∗∗*p* < 0.01 *versus* BioID2 by ANOVA. HA, hemagglutinin; HRP, horseradish peroxidase.

We next compared the labeling efficiency of the MicroID2 and lbMicroID2 constructs to the original BioID2 and other biotin ligases. Of the recently described “abortive” biotin ligases, TurboID and miniTurbo demonstrated the highest labeling activity (11). We therefore overexpressed BioID2, MicroID2, lbMicroID2, miniTurbo, and TurboID either on their own (Fig. 6, *A* and *B*) or fused to the transmembrane protein MARCH1 (Fig. 6*C*). MicroID2 had similar background labeling prior to the addition of biotin as BioID2; however, MicroID2 had considerably higher total labeling (Fig. 6, *A*–*C*). In addition, although it is the smallest biotin ligase yet described (180 AA for MicroID2 *versus* 257 AA for miniTurbo and 338 AA for TurboID), MicroID2 was only slightly less active than TurboID and considerably more active than miniTurbo (Fig. 6, *A*–*C*). Finally, compared with BioID2, the lbMicroID2 mutant had lower levels of background labeling in the absence of exogenous biotin while showing enhanced total labeling (Fig. 6, *A* and *C*). We subtracted background labeling from labeling observed after the addition of biotin to determine the biotin-stimulated labeling efficiency. In this comparison, TurboID and MicroID2 were indistinguishable, and both lbMicroID2 and miniTurbo were considerably more active then BioID2 (supplemental Fig. S5, *A* and *B*).

We also examined whether MicroID2 could label at short intervals demonstrated previously with TurboID and miniTurbo (11). Although short-term labeling was more variable than observed at longer time points, we observed an increase in labeling with TurboID and miniTurbo over 10 min. In our hands, MicroID2 resulted in a higher labeling when normalized for expression (Fig. 6*B*). These data suggest that both MicroID2 and lbMicroID2 possess considerable advantages over the original BioID2 construct and that each construct provides unique benefits depending on the proposed experiments.

### Proteomics of Subcellular Organelles With MicroID2

**Fig. 7:**
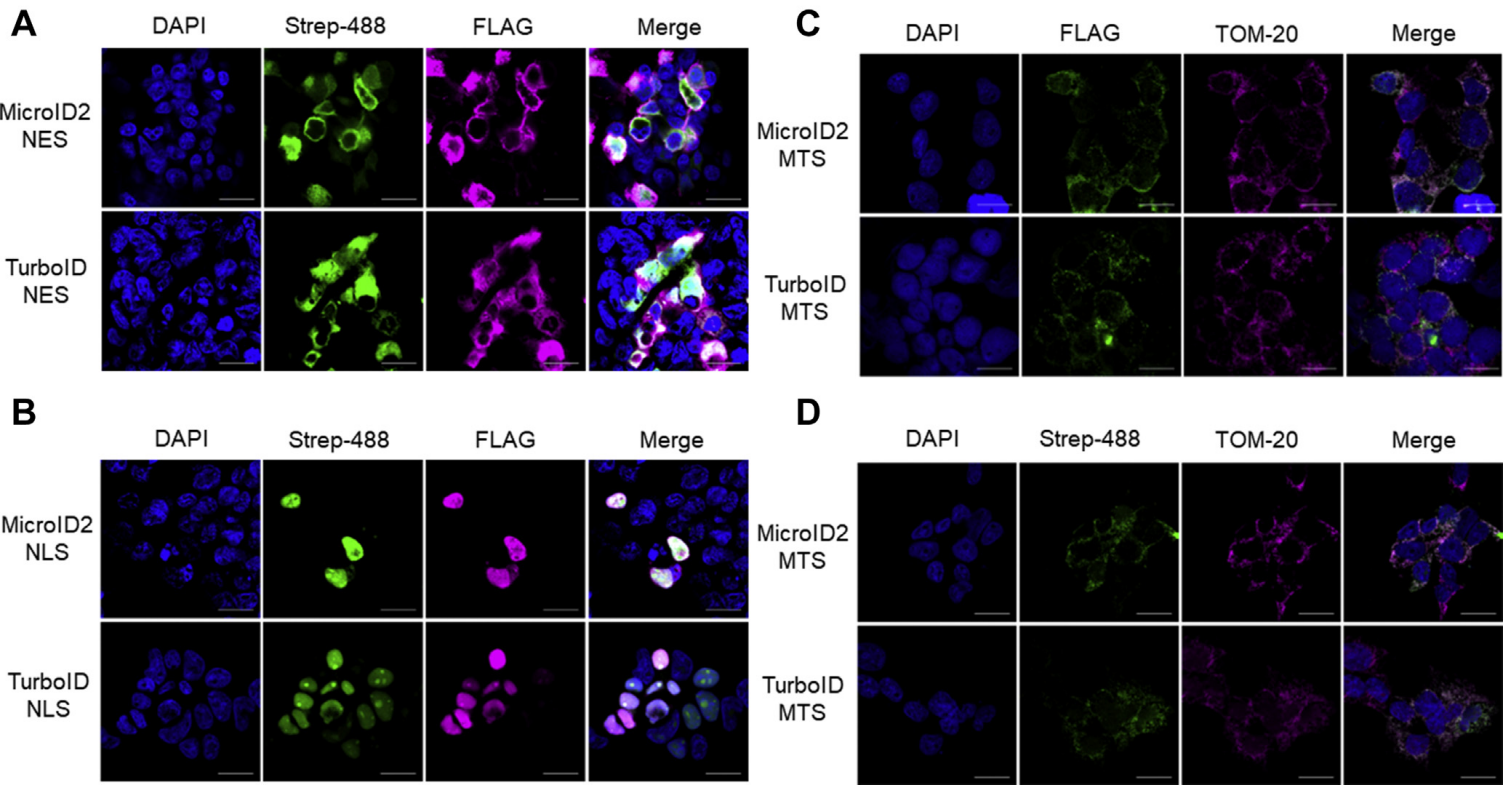
**Localization of targeted MicroID2 constructs.** We added subcellular localization motifs to N-terminal and C-terminal domains of MicroID2 and TurboID constructs (NES, NLS, and MTS). We transiently transfected human embryonic kidney (HEK) cells with MicroID2 and TurboID constructs and performed immunofluorescence to measure subcellular localization. *A*, NES localization: Streptavidin-488 = biotinylation; MicroID2/TurboID expression; FLAG = MicroID2/TurboID expression. *B*, NLS localization: streptavidin-488 = biotinylation; FLAG = MicroID2/TurboID expression. *C*, MTS localization: FLAG = MicroID2/TurboID expression; TOM20 = mitochondrial outer membrane marker. *D*, MTS localization: Streptavidin-488 = biotinylation; TOM20 = mitochondrial outer membrane marker. *A*–*D*, the scale bar represents 20 μm. Representative of two independent experiments. MTS, mitochondrial targeting sequence; NES, nuclear export sequence; NLS, nuclear localization sequence.

The isolation of subcellular organelles is technically challenging, and preparations frequently contain contaminants. Targeting a proximity enzyme to a specific organelle/subcellular compartment using localization motifs simplifies identification of changes in the subcellular compartments compared with density or chemical-based isolation and purification. To determine the utility of MicroID2 in subcellular proteomic analysis, we localized both TurboID and MicroID2 to three subcellular compartments. We appended the NES to the C-terminal domain to label cytosolic proteins, the 3× NLSs to the N terminus (18) to label nuclear proteins, and the 2× MTS to the N-terminal domain to measure mitochondrial proteins (19). After cloning, we transiently transfected the constructs into human embryonic kidney cells and examined localization *via* immunofluorescence microscopy. We measured expression of both MicroID2 and TurboID constructs in the cytoplasm (Fig. 7*A*), nucleus (Fig. 7*B*), and the mitochondria (Fig. 7, *C* and *D*) when specifically localized to these regions. The addition of exogenous biotin also resulted in specific biotinylation at the localization site (Fig. 7, *A*, *B* and *D*).

**Fig. 8:**
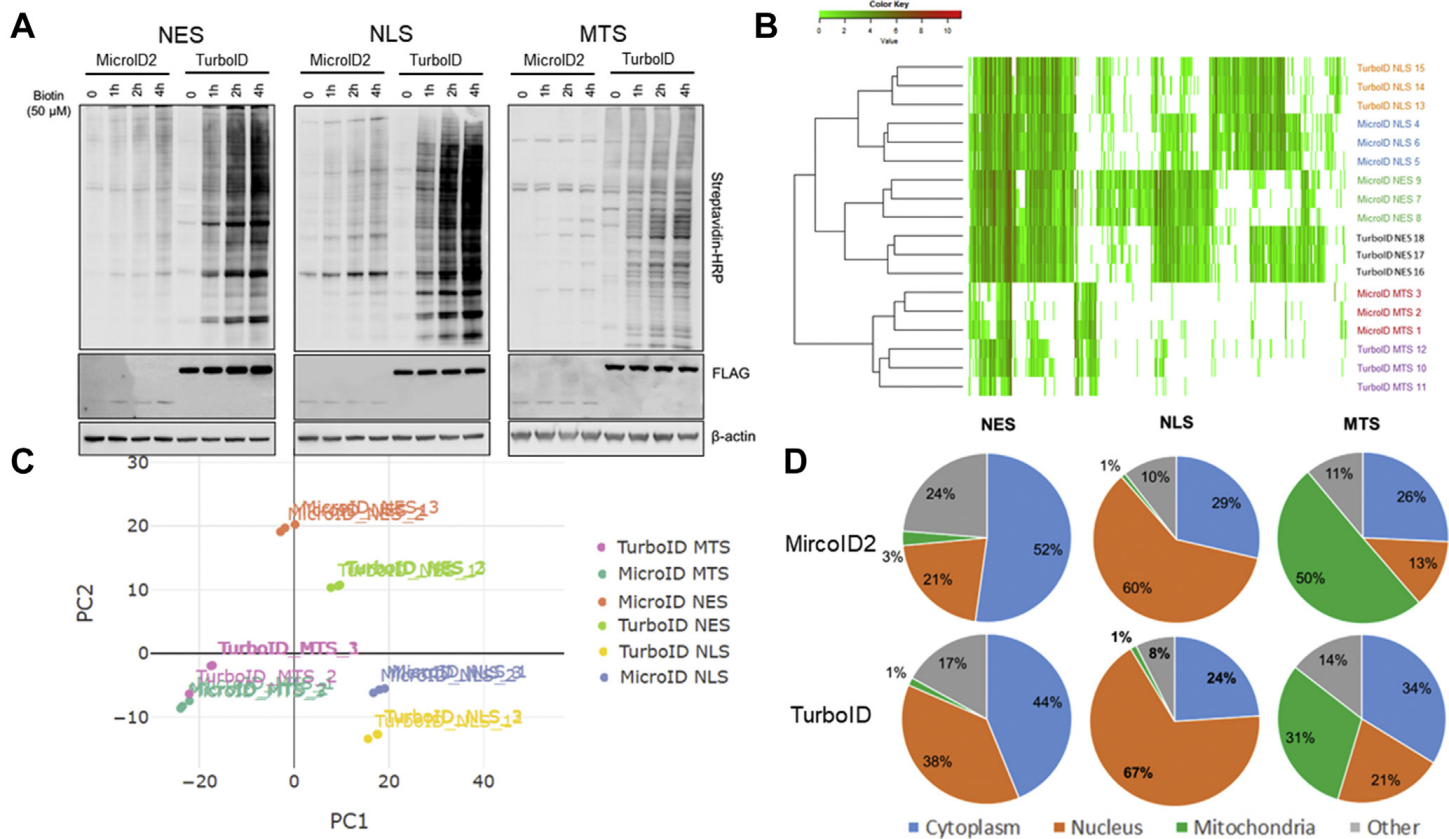
**Proteomics of subcellular organelles with MicroID2.***A*, biotinylation activity of stably integrated MicroID2 and TurboID constructs. Following stable integration of the biotin ligase constructs using the sleeping beauty transposon system, we treated cells with biotin for the indicated times. After cell lysis, we performed Western blotting to examine MicroID/TurboID construct expression (FLAG) and biotinylation (streptavidin-488). Representative of two independent experiments. *B*, heat map of all proteins identified using the Prostar proteomics GUI (26). *C*, MDS plot was generated from the top 500 proteins identified using the TCC GUI package (27). *D*, stable MicroID2 and TurboID BEAS-2B cell lines were treated with exogenous biotin for 3 h (MicroID2) or 1 h (TurboID). After harvesting the cells, we pulled down biotinylated proteins with streptavidin–HRP and submitted for mass spectrometry. Un-normalized spectral counts present in at least two samples and averaging to three peptides per group were included in the analysis. Unique proteins identified and mapped to subcellular organelles: MicroID-NES = 1639; MicroID-NLS = 1686; MicroID-MTS = 334; TurboID-NES = 1700; TurboID-NLS = 1635; and TurboID-MTS = 379. n = 3 samples per group. UniProt IDs were utilized to assign subcellular location using the “Retrieve/ID Mapping” tool from the UniProt database. GUI, graphical user interface; HRP, horseradish peoxidase.

We then stably integrated all constructs into the lung epithelial cell line BEAS-2B using the sleeping beauty transposase system (20, 21). We confirmed MicroID2 and TurboID construct expression by measuring FLAG and examined the extent of biotinylation with streptavidin–HRP (Fig. 8*A*). MicroID2 expressed at lower levels than TurboID suggesting a decrease in stability. This is consistent with earlier findings for miniTurbo and may be due to the smaller size or truncated nature of these constructs (12). Interestingly, transient transfection of these constructs did not lead to inherent differences in expression suggesting that stable integration was responsible for the lower expression observed (supplemental Fig. S6*A*). To test if this instability was seen with other constructs, we stably integrated some of the constructs tested *via* transient transfection in Figure 6. We observed similar instability with MicroID2-HA *versus* TurboID-HA in BEAS-2B (supplemental Fig. S6*B*). We also observed greater instability with the MARCH1-MicroID2-HA *versus* MARCH1-TurboID-HA in BEAS-2B cells although to a lesser extent (supplemental Fig. S6*C*). Overall labeling with the localized constructs was sufficiently robust to perform proteomics in subcellular organelles. We therefore seeded MicroID2 and TurboID constructs into 10 cm dishes and labeled with biotin for 1 h (TurboID) or 3 h (MicroID2) to examine biotinylation at comparable levels (12). Following biotinylation, we isolated biotinylated proteins with streptavidin beads (supplemental Fig. S7*A*) and analyzed them by MS. Relative protein quantitation is measured by comparing the number of MS/MS spectra identified from the same protein across samples. Unnormalized spectral counts of the proteins identified were visualized by heat map (Fig. 8*B*). We also quantified the similarity of each construct to each other and to each replicant *via* principal component analysis (Fig. 8*C*). Subcellular localized constructs identified highly similar proteins regardless of which biotin ligase was appended with a high degree of reproducibility for both ligases. We then compared cellular localization of peptides identified with each construct using Gene IDs assigned to subcellular locations using the UniProt “Retrieve/ID Mapping” tool. Compared across groups, NES enriched the identification of cytosolic proteins, NLS enriched nuclear proteins, and the MTS construct enriched mitochondrial proteins. Importantly, both MicroID2 and TurboID demonstrated a similar enrichment indicating both constructs have a similar capability of specifically labeling subcellular organelles (Fig. 8*D*). We also compared the proteins identified with each construct to determine the degree of overlap. Similar to the principal component analysis, the MicroID2/TurboID constructs localized to the same cellular compartment had a higher degree of similarity (supplemental Fig. S7*B*) than MicroID2 constructs localized to different compartments (supplemental Fig. S7*C*). These results demonstrate that the MicroID2 biotin ligase is a useful tool for proximity-dependent biotinylation proteomics in subcellular organelles.

## Discussion

Several recently developed biotin ligases facilitate the measurement of PPIs including BioID2. BioID2 was initially developed as a smaller alternative to the BirA of *E. coli* BioID and has been utilized in experimental conditions to identify interacting proteins (9, 22, 23, 24). Here, we report that truncation of the C terminus improved BioID2 labeling, whereas decreasing background biotinylation. By performing targeted site-directed mutagenesis based on the crystal structure of the BirA R40G mutant of *A. aeolicus*, we were able to increase the biotinylation efficiency of this construct. Previous studies examining the structure of *A. aeolicus* show that although the R40G mutant has little effect on biotin binding, this mutation leads to an altered confirmation in the ATP-binding pocket, resulting in lower ATP binding (17). Since isothermal calorimetry analysis demonstrates that *A. aeolicus* binds biotin and ATP randomly in a cooperative process, we hypothesize that our mutations increase access to the ATP-binding site leading to greater overall biotinylation activity. We also speculated that the C-terminal region plays a larger role in biotin scavenging than ATP binding since biotinylation activity remains high in the Δ63 mutant. Interestingly, the *E. coli* mutant BirA R118G has significantly reduced biotin binding compared with wildtype BirA (17). This may explain why deletion of the C-terminal domain is more detrimental to the function of miniTurbo than BioID2.

The majority of the first-generation ligases have very low kinetics that stipulate labeling times of about several hours to overnight (2, 3). Yeast-directed evolution yielded the faster labeling of biotin ligases, TurboID, and miniTurbo. MicroID2 demonstrates enhanced biotin labeling over first-generation ligases, and despite being half the size of TurboID, MicroID2 has only slightly reduced labeling efficiency. When compared with miniTurbo, MicroID2 is one-third smaller with increased labeling. During the execution of these studies, we became aware of a preprint independently demonstrating that the C terminus of BioID2 is dispensable and that mutation of the L41 residue increased labeling efficiency (25). This construct is similar to the truncated construct developed in this article. We in addition demonstrate further mutations that led to a greater enhancement in activity and lower background. While performing these studies, we observed that miniTurbo had lower background labeling than MicroID2. To increase the utility of MicroID2, we also developed a less active MicroID2 mutant (lbMicroID2) that has significantly reduced background labeling for situations where biotin scavenging would be problematic, such as in *in vivo* studies and in cell culture conditions that require biotin supplementation.

One potential drawback to the MicroID2 construct was the observed apparent instability demonstrated when stably integrated. This instability was also demonstrated with miniTurbo when stably integrated into the cell line A549 (12). Interestingly, we did not observe consistent instability with transient transfection with either MicroID2 or miniTurbo. In future studies, we will determine if these inherent differences in stability were unique to the sleeping beauty system or are conserved with other integration systems. Although there were stability in differences, we were able to compare proximity-dependent biotinylation of MicroID2 and TurboID by localizing each construct to subcellular organelles and measuring biotinylated proteins by MS proteomics. We had comparable localization and proteomics profiles for both constructs suggesting MicroID2 is a useful alternative to TurboID, especially when a smaller construct is desirable. Although additional testing is necessary, certain subcellular organelles may be more amenable to labeling with MicroID2 *versus* larger biotin ligases because of steric hindrances. Importantly, these studies showed a greater degree of agreement between substrates labeled by MicroID2 and TurboID constructs localized to a specific organelle than with the same construct localized to a different organelle. This demonstrated that the localization of the ligase had a larger impact on proteins identified as opposed to the origin of the BirA under these conditions.

In summary, we developed a small biotin ligase that facilitates rapid labeling of proximal proteins. We demonstrated that this construct is both smaller with greater biotinylation activity than BioID2. In addition, it facilitates more robust labeling than the smaller miniTurbo, the most active currently described small biotin ligases. Finally, we demonstrated that MicroID2 could be localized to subcellular organelles to facilitate proximity-labeling proteomics studies. MicroID2 and lbMicroID2 have considerable advantages over previously developed biotin ligases including the smaller size and lower background labeling.

## Data Availability

The MS proteomics data have been deposited to the ProteomeXchange Consortium *via* the PRIDE [1] partner repository with the dataset identifier PXD031146 and 10.6019/PXD031146. The detailed project information is as follows:

Project name: MicroID2: A novel biotin ligase enables rapid proximity-dependent proteomics. Project accession number: PXD031146. Project DOI: 10.6019/PXD031146

Plasmids developed in this article including MicroID2 and lbMicroID2 will be deposited to the Addgene plasmid repository.

## Supplemental data

This article contains supplemental data.

## Conflict of interest

The authors declare that they have no conflicts of interest with the contents of this article.

## Supplemental Data

MicroID Supplemental

Unnormalized Spectal Counts; MicroID vs. TurboID

Background contaminants in MIcroID2 and TurboID
