# Supplementary material for: MicroID2: A Novel Biotin Ligase Enables Rapid Proximity-Dependent Proteomics
Source: Mol Cell Proteomics. 2022 Jun 8;21(7):100256. doi: 10.1016/j.mcpro.2022.100256 (PMC9293651; doi:10.1016/j.mcpro.2022.100256)
Supplement: MicroID Supplemental [file mmc1.pdf]

A)

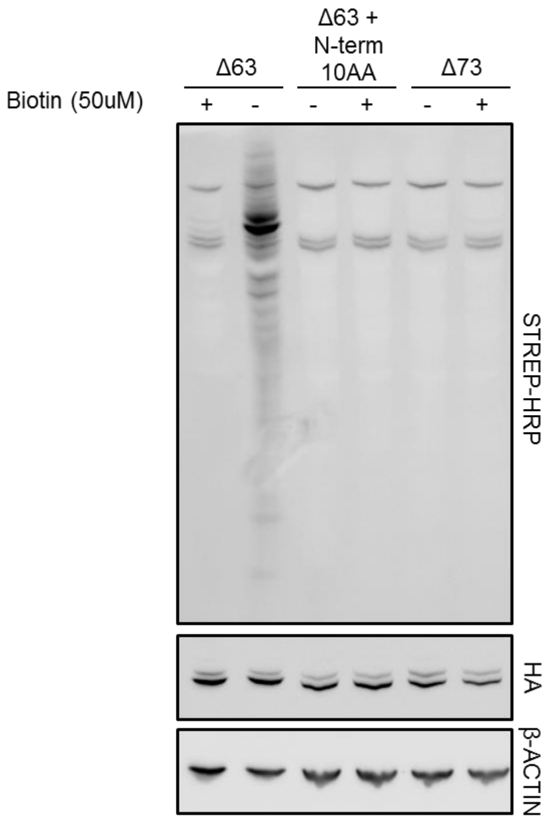

Fig. S1. N-terminal truncation and additional c-terminal truncations completely inactivate Δ63 truncated BioID2. HEK cells were transfected with IFNLR1-BioID2 mutants: Δ63, Δ63 + 10AA N-terminus deletion, and Δ73. At 48 hours post-transfection, cells were labeled for 1 hour with biotin at the indicated concentrations and probed with Streptavidin-HRP to measure biotinylation and HA to measure biotin ligase expression..

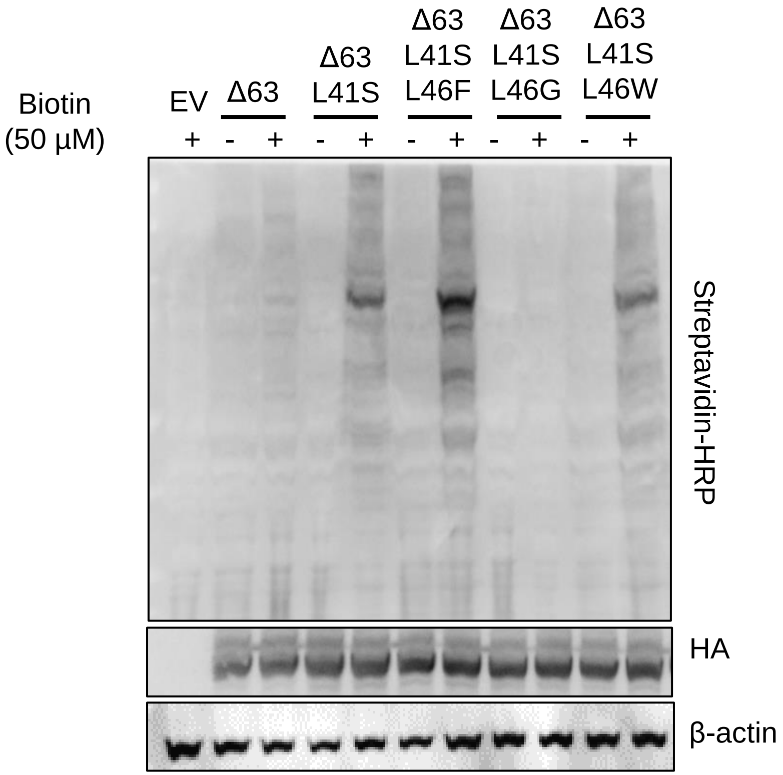

Fig. S2. Examining substitution at L46. HEK cells were transfected with IFNLR1-BioID2 mutants: Δ63, Δ63-L41S, Δ63-L41S-L46F, Δ63-L41S-L46G, Δ63-L41S-L46W. At 48 hours post-transfection, cells were labeled for 2 hours with biotin at the indicated concentrations and probed with Streptavidin-HRP to measure biotinylation and HA to measure biotin ligase expression..

Fig. S3

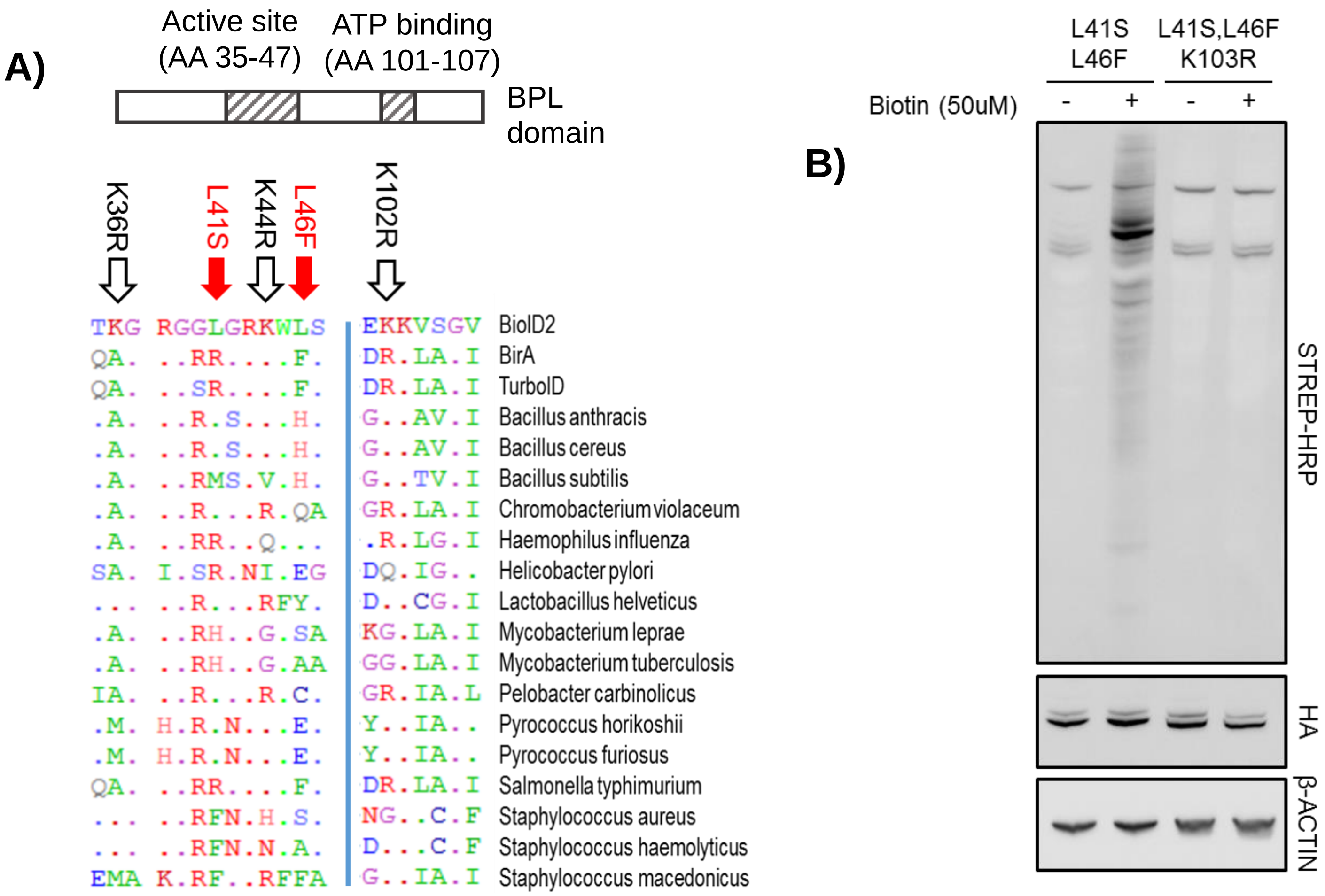

Fig. S3. Substitution of lysine residues in the active site moderately increases biotin labeling. A) Alignment of the active site of BioID2 (AA 35-47) and the active site adjacent (AA 101-107) region using the GENtle software. Outline Arrow: previous mutations from figure 3. Solid Arrow: sites mutated in these experiments. **B)** HEK cells were transfected with IFNLR1-Δ63-L41S-L46F and Δ63-L41S-L46G-K103R. At 48 hours post-transfection, cells were labeled for 1 hour with biotin at the indicated concentrations and probed with Streptavidin-HRP to measure biotinylation and HA to measure biotin ligase expression.

Fig. S4

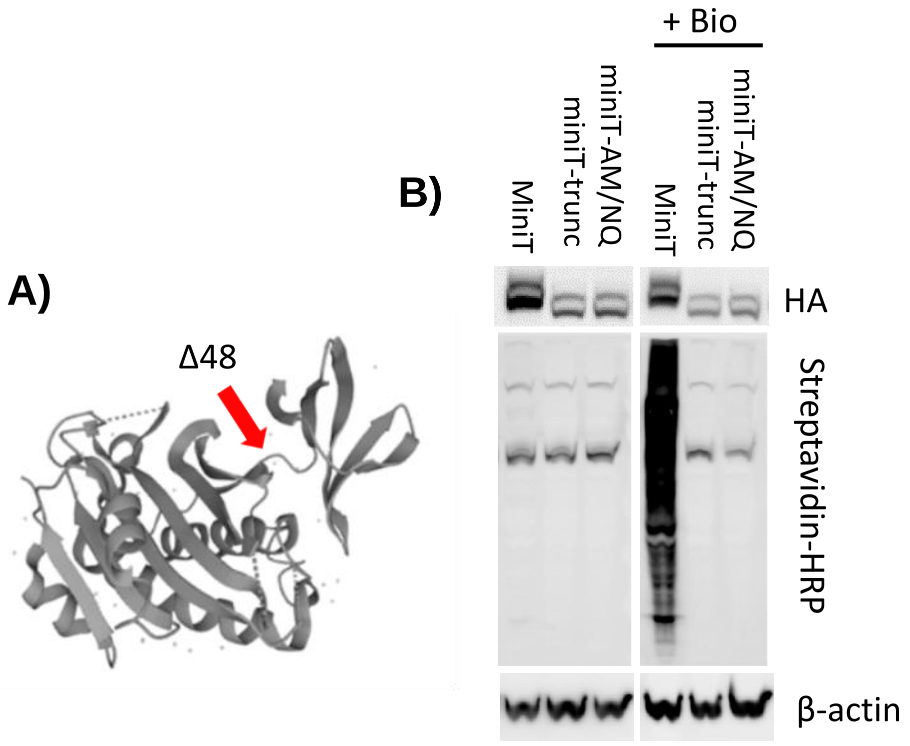

Fig. S4. MiniTurbo requires the c-terminal domain for activity A) Crystal structure of *E. coli* BirA (1BIA) with the n-terminal domain removed to resemble the miniTurbo structure. https://www.uniprot.org/uniprot/P06709 Red Arrow: truncation site to remove c-terminal domain. **B)** HEK cells were transfected with IFNLR1-miniTurbo, truncated IFNLR1-minTurbo, and truncated IFNLR1-miniTurbo with mutations to resemble MicroID2 (AM/NQ). At 48 hours post-transfection, cells were labeled for 1 hour with biotin at the indicated concentrations and probed with Streptavidin-HRP to measure biotinylation and HA to measure biotin ligase expression.

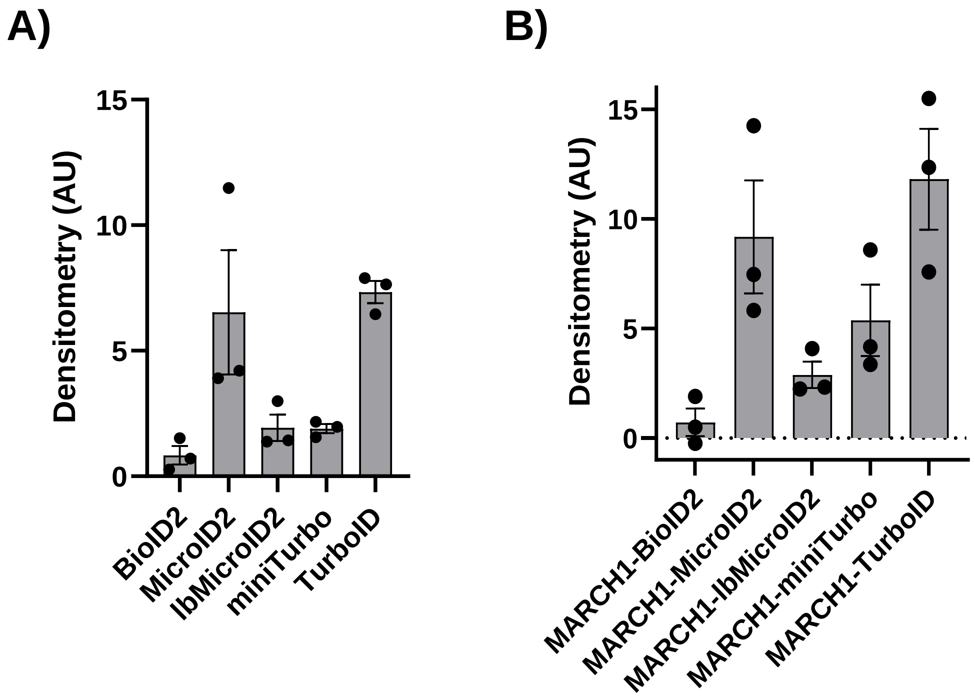

Fig. 6. Comparison of biotin ligase activity. HEK cells were transfected with several biotin ligases including BioID2, MicroID2, lbMicroID2 (containing additional I120A, N125A mutants), miniTurbo, and TurboID. Constructs were either expressed alone or conjugated to the transmembrane protein MARCH1. Biotin labeling was quantified after subtracting background biotinylation to determine to total biotin-stimulated labeling in biotin ligase alone **A)** and appended to MARCH1 **B)** conditions from figure 6.

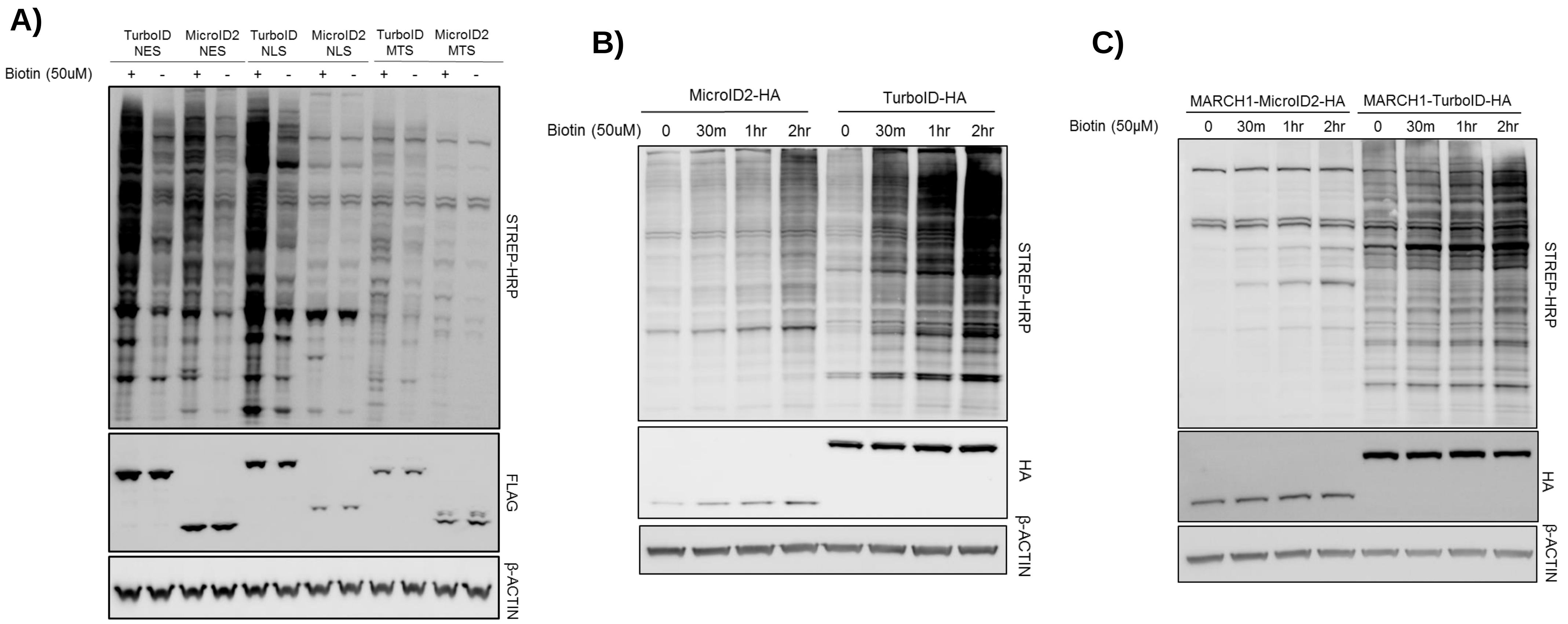

Fig. S6. Examining MicroID2 stability. A) Transient transfection of biotin ligases MicroID2 and TurboID appended to localization sequences (Nuclear export sequence = NES, nuclear localization sequence = NLS, mitochondrial targeting sequence = MTS). After cell lysis, we performed western blotting to examine MicroID/TurboID construct expression (FLAG) and biotinylation (streptavidin-HRP). **B, C)** Biotinylation activity of stably integrated MicroID2 and TurboID constructs either alone **B)** or appended to MARCH1 **C)** following stable integration of the biotin ligase constructs using the sleeping beauty transposon system, we treated cells with biotin for the indicated times. After cell lysis, we performed western blotting to examine MicroID/TurboID construct expression (HA) and biotinylation (streptavidin-HRP).

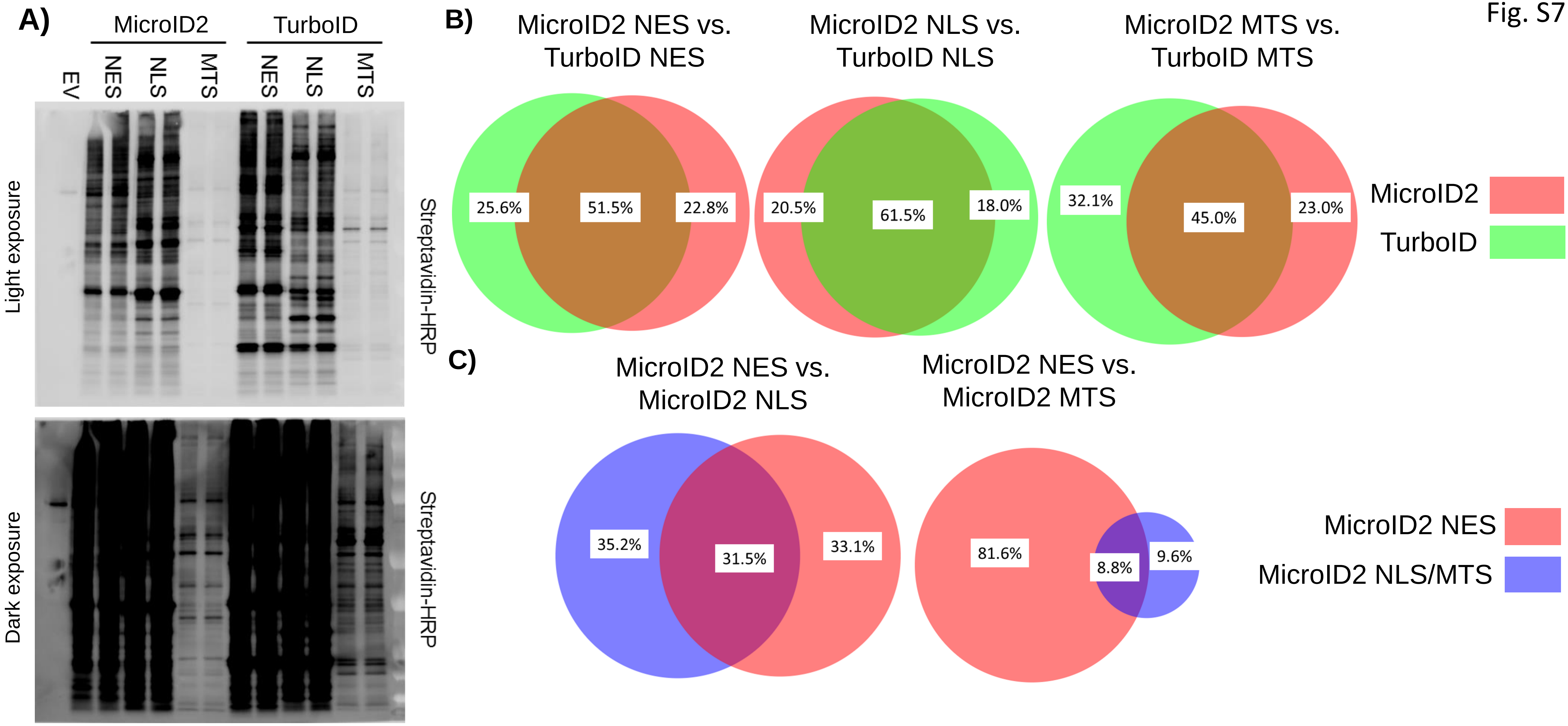

Fig. S7. Streptavidin PD. A) Wild-type BEAS2B cells, or cells stably expressing TurboID or MicroID2 targeted to the cytosol (NES), nucleus (NLS) or the mitochondria (MTS) were seeded into 10 cm dishes. At 24 hours post-seeding, cells were treated with 50 µM biotin for 3h (MicroID2) or 1h (TurboID) followed by isolation of total protein and pulldown of biotinylated proteins with streptavidin beads. 10% of the total volume was loaded onto an SDS-PAGE gel to confirm isolation of biotinylated peptides prior to mass spectrometry analysis. We analyzed biotinylated proteins from MicroID2 and TurboID constructs localized to the cytoplasm (NES), nucleus (NLS), and the mitochondria (MTS) (n=3 samples per group). **B)** Percent overlap of proteins identified from MicroID2 and TurboID constructs. **C)** Percent overlap of proteins identified from MicroID-NES, MicroID-NLS, and MicroID-MTS. **B&C)** Graph generated using: https://www.deepvenn.com. Peptides present in at least 2 samples and averaging to 3 peptides per group were included in the analysis. n=3 samples per group.
